# Medical Opinion and Movement

**Published:** 1906-11-17

**Authors:** 


### Medical Opinion and Movement.

Whatever may be the ultimate result, the thanks of general practitioners are due to the Hospitals Committee of the British Medical Association for the active propaganda they have instituted in support of a remedy for the present hospital abuse. Whatever may be the view held as to the value of the suggestions this committee have made, there can be no doubt that their proceedings and persistence have brought the question of hospital abuse into the foreground of public discussion, and that is a service which we must all be grateful to them for rendering.

The question of hospital abuse has of course more than one side. We dealt with the medical side of it, mainly, in a leading article in our columns last week. A consultant in active practice sends us an interesting communication, which confirms the view we advanced, for it rested on the feeling, that at the bottom, few, if any, of those immediately responsible for the ever increasing amount of free medical relief at present given by the hospitals, have any burning desire to reduce it. The consultant, in question, properly points out, as he sees the facts, that "the out-patient departments, and indeed hospital attendances as a whole, have been encouraged by the directors of the hospitals generally, and by the secretaries, the ever-increasing number of patients being used as levers to work the charitable public." This, we think, was much more the case formerly, in London at any rate, than it is to-day. Our correspondent writes, however, "Even to-day, as you know, Mr. Sydney Holland, the lay representative of one of the largest hospitals, not only defends, but is proud of, his enormous out-patient department in Whitechapel." He is therefore unconvinced, and naturally desires, as a consultant, not to have pressure brought to bear upon himself and his colleagues by the general practitioners—the course we ventured to urge last week. Of course the visiting staffs have their difficulties, and it is essential to also secure the whole-hearted co-operation of the committees of management, and the lay officials of hospitals, but the key to the position is held by the visiting staffs, and if they can be got to move energetically, we have little doubt that a large reduction in the present volume of free medical relief given by London hospitals, will spedily be made. To effect this, it is essential, that all interested should co-operate, energetically, with a sincere desire to abolish hospital abuse, and to secure the restriction of free medical relief to those patients alone who are clearly entitled to claim it.

An effectual remedy will only be forthcoming through such co-operation, based upon a just appreciation of the difficulties which underlie the position occupied by the visiting medical staffs, the hospital committees, and the general practitioners, respectively. Our correspondent puts the case of the medical staffs tersely and well: "The movement to modify this state of matters (*i.e.* the excessive free medical relief) is very largely provided by the medical profession in the shape, for example, of the Hospitals Committee of the British Medical Association, and a steady attempt is being made, and in my own hospital the physicians and surgeons are taking their full share, to enlighten the lay directors on the evils of the present system. I myself have been fighting for two years to get a hospital committee to do away with the absurdity of the hospital letter system. In view of these circumstances, I cannot consider it fair to suggest, that the medical officers of hospitals are the persons responsible for the existing conditions, and, as I said before, I cannot view with sympathy the suggestion, that the general practitioners should combine for the purpose of exercising pressure on the medical staffs of the hospitals. It is the lay not the medical authorities that need such pressure." We are inclined to agree with our correspondent, that pressure must be put upon both the lay and the medical authorities of hospitals, to reduce the excessive free medical relief at present given by these institutions. The small results from the attempts at reform, which have been made during the last thirty years, prove to demonstration, in our judgment, however, that until pressure is brought to bear on the visiting medical staffs, by the general practitioners, with sufficient energy to make them realise that this question cannot longer remain in its present position, but must be faced and changed through the action of the medical staffs, and in co-operation with the lay authorities, nothing effective in the way of reform is ever likely to take place.

It is becoming more and more evident that practitioners in the country, and those with considerable practices in towns, are realising the many advantages which a good motor offers over horse traction. Those members of the profession who have any mechanical instinct have probably long ago purchased their motor, and are using it with increasing comfort and satisfaction as they perfect their knowledge of its possibilities and requirements. Experience seems to indicate that those owners who have caused their coachmen to be trained as chauffeurs obviate many difficulties. The motor exhibition at Olympia which is now proceeding affords the best possible means to see the enormous developments which have taken place and the infinite variety of motors from which a selection may be made. The novice need have no fear nowadays of being lost in a maze of machinery. Firms of engineers gladly offer him their gratuitous services as guides, and will demonstrate the advantages which one machine offers over another, and the kind of motor best suited to fulfil the requirements of each customer. It is a great point gained to understand the mechanism of one's own motor, for such knowledge leads to a large reduction in the cost of upkeep each year. Its possession is not difficult of attainment, and is well worth the outlay it represents.

